# Bacterial findings in patients referred to hospital for the treatment of acute tonsillitis with or without peritonsillar phlegmon

**DOI:** 10.1186/s12879-023-08420-8

**Published:** 2023-06-29

**Authors:** Camilla Andersen, Thomas Greve, Kasper Basse Reinholdt, Ann Marlene Gram Kjaerulff, Nichlas Udholm, Vesal Khalid, Adnan Madzak, Christophe Duez, Henrik Münch, Søren Pauli, Christian Sander Danstrup, Niels Krintel Petersen, Tejs Ehlers Klug

**Affiliations:** 1 Department of Clinical Microbiology, Aarhus University Hospital, Palle Juul-Jensens Boulevard 99 Aarhus N, Aarhus, DK-8200 Denmark; 2 Department of Otorhinolaryngology, Head & Neck Surgery, Aarhus University Hospital, Aarhus, Denmark; 3 Department of Otorhinolaryngology, Head & Neck Surgery, Aalborg University Hospital, Aalborg, Denmark; 4 Aarhus University, Aarhus, Denmark

**Keywords:** *Streptococcus pyogenes*, *Fusobacterium necrophorum*, *Streptococcus dysgalactiae*, Dysbacteriosis

## Abstract

**Background:**

The vast majority of patients with acute tonsillitis (AT) are managed in general practice. However, occasionally patients are referred to hospital for specialized management because of aggravated symptoms and/or findings suggestive of peritonsillar involvement. No prospective studies have been conducted aiming to investigate the prevalent and significant microorganisms in this highly selected group of patients. We aimed to describe the microbiological findings of acute tonsillitis with or without peritonsillar phlegmon (PP) in patients referred for hospital treatment and to point out potential pathogens using the following principles to suggest pathogenic significance: (1) higher prevalence in patients compared to healthy controls, (2) higher abundance in patients compared to controls, and (3) higher prevalence at time of infection compared to time of follow up.

**Methods:**

Meticulous and comprehensive cultures were performed on tonsillar swabs from 64 patients with AT with (n = 25) or without (n = 39) PP and 55 healthy controls, who were prospectively enrolled at two Danish Ear-Nose-Throat Departments between June 2016 and December 2019.

**Results:**

*Streptococcus pyogenes* was significantly more prevalent in patients (27%) compared to controls (4%) (*p* < 0.001). Higher abundance was found in patients compared to controls for *Fusobacterium necrophorum* (mean 2.4 vs. 1.4, p = 0.017) and *S. pyogenes* (mean 3.1 vs. 2.0, p = 0.045) in semi-quantitative cultures. *S. pyogenes, Streptococcus dysgalactiae, and Prevotella* species were significantly more prevalent at time of infection compared to follow up (*p* = 0.016, *p* = 0.016, and *p* = 0.039, respectively). A number of species were detected significantly less frequently in patients compared to controls and the mean number of species was significantly lower in patients compared to controls (6.5 vs. 8.3, p < 0.001).

**Conclusions:**

Disregarding *Prevotella* spp. because of the prevalence in healthy controls (100%), our findings suggest that *S. pyogenes*, *F. necrophorum*, and *S. dysgalactiae* are significant pathogens in severe AT with or without PP. In addition, infections were associated with reduced diversity (dysbacteriosis).

**Trial registration:**

The study is registered in the ClinicalTrials.gov protocol database (# 52,683). The study was approved by the Ethical Committee at Aarhus County (# 1-10-72-71-16) and by the Danish Data Protection Agency (# 1-16-02-65-16).

**Supplementary Information:**

The online version contains supplementary material available at 10.1186/s12879-023-08420-8.

## Introduction

Acute tonsillitis (AT) is a very frequent reason for consultation in general practice [1]. The majority of patients have uncomplicated, self-limiting disease and do not require antibiotic treatment [2]. *Streptococcus pyogenes* is considered the prevalent bacterial cause of AT [3]. In Denmark and most of the world, it is common only to investigate for the presence of *S. pyogenes* in throat swabs and often only by the use of a rapid antigen detection test [4]. However, studies also suggest that *Streptococcus dysgalactiae*, *Arcanobacterium haemolyticum* [5], and (more recently) *Fusobacterium necrophorum* may be important pathogens in AT [6–8].

The vast majority of patients with AT are managed in general practice, but approximately 300 patients are annually referred to the Ear-Nose-Throat Departments at Aarhus University Hospital and Aalborg University Hospital for more specialized treatment. Hence, referred patients constitute a highly selected subgroup of patients with AT either with severe symptoms or signs of peritonsillar phlegmon (PP). This subgroup of patients with severe throat infections are treated with antibiotics without prior microbiological investigations as they are considered to be bacterially infected. A few studies of hospitalized patients with AT describe the bacteriological findings in routine cultures [9–11], but no prospective studies have been conducted aiming to investigate the prevalent and significant microorganisms in this highly selected group of patients with severe AT.

The current study was undertaken to: (1) describe the microorganisms found in AT with or without PP referred for treatment in a hospital setting using meticulous and comprehensive aerobic and anaerobic culture methods, and (2) investigate which of the cultured microorganisms are likely pathogens.

## Materials and methods

### Participants

#### Patients

Patients were prospectively enrolled in the period June 2016 - December 2019 at two Danish Departments of Otorhinolaryngology, Head and Neck Surgery (Aarhus University Hospital and Aalborg University Hospital). The inclusion criteria were: (1) patients with AT with or without PP referred for hospitalization or outpatient treatment, (2) age 15–40 years, (3) Centor Score 3–4, and (4) written and oral consent. The exclusion criteria were: (1) peritonsillar abscess (PTA), (2) acute or previous tonsillectomy, and (3) biochemical signs of infectious mononucleosis. No screening log for in- and exclusion of patients were performed.

AT was defined as anamnestic sore throat and pain on swallowing in addition to visual inflammation of the tonsillar mucosa. PP was defined as the clinical finding of swelling of the peritonsillar tissue without detection of peritonsillar pus. PTA was defined as visual detection of peritonsillar pus.

#### Healthy controls

With an aim to pinpoint the likely pathogens in AT, healthy controls (medical students) fulfilling the following criteria were enrolled in the period March - May 2022 as reference group: (1) age 15–40 years, (2) no symptoms or signs of acute throat infection within the last three months, (3) no more than three throat infections within the last two years, (4) no antibiotic treatment within the last three months, and (5) no previous tonsillectomy.

### Sample collection

#### Patients

At the time of acute examination, a tonsillar swab (E-swab (Copan, Brescia, Italy)) from both tonsillar surfaces and blood samples for biochemical analysis were obtained. Patients were invited to a follow up examination two to four weeks later, where another tonsillar swab was obtained. The tonsillar swabs were placed at -80 °C within 30 min of collection.

#### Healthy controls

Tonsillar swabs were obtained and processed in the same way as for the patients.

### Microbiological analyses

Samples were processed in a class 2 laminar air flow safety cabinet using an aseptic technique. Swabs were vortexed for 5 s. and the flocked swab was discharged. 10 µL of liquid medium was plated on 5% horse blood agar, chocolate agar, Mueller Hinton agar with 5% horse blood and 20 mg/L NAD plus selected antimicrobial discs used for initial differentiation of bacterial species, anaerobic agar (chocolate plate containing K-vitamin and cysteine), and selective *Fusobacterium* agar (containing 5 mg/L nalidixic acid and 2.5 mg/L vancomycin) (Statens Serum Instutite Diagnostica, Hillerød, Denmark). Plates were incubated at 35 °C, the first three plates in 5% CO_2_, and the latter two plates in anaerobic atmosphere including a metronidazole-disc (10 UG) (Oxiod, Roskilde, Denmark). All plates were incubated for 4 days, but also checked for growth after 2 days. Cultured microorganisms were initially identified to genus-level by phenotypic appearance and biochemical profiles according to accepted guidelines [12] and for several bacteria matrix-assisted laser desorption/ionization time-of-flight mass spectrometry (MALDI-TOF MS) (Bruker Daltonics, Bremen, Germany) was used to obtain species-level identification (see Additional file 1 in supplementary).

Species differentiation between the closely related *Streptococcus anginosus*, *Streptococcus intermedius*, and *Streptococcus constellatus* is inadequate by phenotypic appearance, MALDI-TOF MS, and 16 S rDNA gene fragment analysis and is thus referred to as *Streptococcus anginosus* group. The initial criteria for anaerobic bacteria were growth in anaerobic atmosphere and sensitivity towards metronidazole. The microorganism colony count was reported semi-quantitatively and divided into the following four different categories according to the quantification; 1: 1 colony, 2: 2–49 colonies, 3: 50–500 colonies, and 4: >500 colonies.

### Criteria for pathogenic significance

The following principles to suggest pathogenic significance of identified microorganisms were used in the present study: (1) higher prevalence in patients compared to healthy controls, (2) higher abundance (semi-quantitative count) in patients compared to controls, and (3) higher prevalence at time of infection compared to time of follow up.

### Statistical analyses

Statistical analyses were performed using the Fisher’s exact test for categorical variables (sex, smoking, prevalence), the Student’s *t*-test for continuous variables (age and number of species), the Kruskal-Wallis test for non-parametric variables (semi-quantitative count), and binomial probability test for comparison of number of isolates at time of infection vs. follow up.

Statistical significance was defined as *p* < 0.05.

### Permissions

The study is registered in the ClinicalTrials.gov protocol database (Unique Protocol ID: 52,683). The study was approved by the Ethical Committee at Aarhus County (# 1-10-72-71-16) and by the Danish Data Protection Agency (# 1-16-02-65-16). Informed consent was obtained from all patients in accordance with the guidelines set by the Danish National Board of Health.

## Results

### Patient characteristics

A total of 64 patients and 55 healthy controls were enrolled in the study. There was no statistically significant difference in sex and age between patients (38% males, mean age 26 years) and controls (51% males, mean age 26 years) (*p* = 0.19, Fisher´s exact test and *p* = 0.44, Student’s *t*-test, respectively). Significantly more patients admitted to smoking (41%) compared to controls (4%) (*p* < 0.001, Fisher´s exact test) (Table 1).

**Table 1:** Clinical and biochemical characteristics of 64 patients with acute tonsillitis with or without peritonsillar phlegmon (PP) and 55 healthy controls

Primary infection	Acute tonsillitis			
	All n = 64	Without PP n = 39	With PP n = 25	Controls n = 55
Males	24 (38%)	12 (31%)	12 (48%)	28 (51%)
Age, mean (SD)	25.7 (5.6)	26.4 (5.4)	24.4 (5.8)	26.3 (1.4)
Duration of symptoms, days median (range)	4.0 (1.0–13.0)	3.0 (1.0–12.0)	5.0 (1.0–13.0)	
Reasons for referral				
Suspected peritonsillar abscess	48 (75%)	23 (59%)	25 (100%)	
Dehydration	14 (22%)	12 (31%)	2 (8%)	
Pain	23 (36%)	18 (46%)	5 (20%)	
Other^1^	6 (9%)	5 (13%)	1 (4%)	
Antibiotic treatment prior to admission	32 (50%)^2^	20 (51%)	12 (44%)	
Tobacco smoking (current)	26 (41%)	18 (47%)	8 (32%)	2 (4%)
Temperature, mean ^o^C (SD)	38.4 (0.8)	38.4 (0.9)	38.3 (0.8)	
Biochemistry, mean (SD)				
C-reactive protein, mg/L	167.0 (112.7)	177.7 (110.5)	150.1 (116.3)	
Leukocyte count, x10^9^/L	14.6 (5.0)	14.0 (5.5)	15.7 (4.0)	
Neutrophil count, x10^9^/L	11.6 (4.7)	10.9 (5.2)	12.7 (3.8)	
Lymphocyte count, x10^9^/L	1.6 (0.7)	1.6 (0.8)	1.5 (0.4)	

^1^ Dyspnoea/upper airway obstruction (n = 3), General malaise (n = 1), Neck swelling (n = 1), Lack of improvement after antibiotic treatment (n = 1)

^2^ Phenoxymethyl-penicillin (n = 29) or amoxicillin-clavulanic acid (n = 3)

Based on clinical findings, 39 (61%) patients were categorized as AT without PP and 25 (39%) patients with PP. In addition to sore throat and pain on swallowing, patient´s symptoms included anamnestic fever (91%) and otalgia (78%). Frequent clinical findings were swollen tonsils (95%), sore cervical lymph nodes (95%), tonsillar exudates (75%), and fever (> 38.0^o^C) (65%). The prevalent reasons for referral to hospital were suspected PTA (73%), pain (36%), and dehydration (22%) (Table 1). Antibiotics were prescribed to 32 (50%) of patients prior to admission; the vast majority (29/32) were treated with phenoxymethyl-penicillin. Thirty-nine (61%) patients were hospitalized for the following reasons: intravenous antibiotic treatment (95%), pain management (72%), rehydration (67%), peritonsillar infection (36%), and/or respiratory distress (5%). The remaining 25 patients (39%) were treated in the outpatient clinic. All patients were treated with antibiotics after admission, either in hospital or as outpatient treatment, of which 59% (38/64) received benzyl-penicillin and 34% (22/64) received phenoxymethyl-penicillin.

### Culture findings

The culture findings in tonsillar swabs from patients and controls are shown in Table 2. The most prevalent isolates in patients were non-hemolytic streptococci (98%), *Prevotella* spp. (83%), *Rothia* spp. (77%), *Corynebacterium* spp. (72%), and *Neisseria* spp. (64%). Similarly, the most prevalent isolates in controls were genera belonging to the commensal pharyngeal flora: non-hemolytic streptococcus (100%), *Prevotella* spp. (100%), *Neisseria* spp. (96%), *Rothia* spp. (82%), *Haemophilus parainfluenzae* (69%), and *Corynebacterium* spp. (56%). The mean number of species cultured from tonsillar swabs obtained from patients (6.5) was significantly lower compared to controls (8.3) (*p* < 0.001, Student’s *t*-test).

**Table 2:** Culture findings in tonsillar surface swabs from 64 patients with acute tonsillitis with or without peritonsillar phlegmon (PP) and 55 healthy controls

Primary infection	Acute tonsillitis							
	All n = 64		Without PP n = 39		With PP n = 25		Controls n = 55	
Microorganisms								
**Aerobic bacteria**								
*Streptococcus pyogenes*		17 (27%)		8 (21%)		9 (36%)		2 (4%)
*Streptocoocus dysgalactiae*		7 (11%)		4 (10%)		3 (12%)		3 (5%)
*Streptococcus anginosus* group		5 (8%)		4 (10%)		1 (4%)		5 (9%)
Non-hemolytic streptococci		63 (98%)		38 (97%)		25 (100%)		55 (100%)
*Haemophilus influenzae*		1 (2%)		1 (3%)				1 (2%)
*Haemophilus parainfluenzae*		16 (25%)		9 (23%)		7 (28%)		38 (69%)
*Haemophilus* spp.		1 (2%)		1 (3%)				13 (24%)
*Staphylococcus aureus*		17 (27%)		10 (26%)		7 (28%)		22 (40%)
Coagulase-negative staphylococci		5 (8%)		4 (10%)		1 (4%)		3 (5%)
*Eikenella corrodens*		8 (13%)		6 (15%)		2 (8%)		2 (4%)
*Neisseria* spp.		41 (64%)		26 (67%)		15 (60%)		53 (96%)
*Corynebacterium* spp.		46 (72%)		26 (67%)		20 (80%)		31 (56%)
*Rothia* spp.		49 (77%)		33 (85%)		16 (64%)		45 (82%)
*Aggregatibacter aphrophilus*								1 (2%)
*Moraxella catarrhalis*		1 (2%)		1 (3%)				
Enterobacteriaceae								1 (2%)
*Gemella* spp.								1 (2%)
*Granulicatella adiacens*		3 (5%)				3 (12%)		
**Anaerobic bacteria**								
*Fusobacterium necrophorum*		7 (11%)		2 (5%)		5 (20%)		5 (9%)
*Fusobacterium* spp.		2 (3%)		2 (5%)				17 (31%)
*Prevotella* spp.		53 (83%)		31 (79%)		22 (88%)		55 (100%)
*Veillonella* spp.		26 (41%)		16 (41%)		10 (40%)		22 (40%)
*Leptotrichia* spp.		5 (8%)		1 (3%)		4 (16%)		25 (45%)
*Lactobacillus* spp.		13 (20%)		8 (21%)		5 (20%)		6 (11%)
*Actinomyces odontolyticus*		11 (17%)		7 (18%)		4 (16%)		20 (36%)
*Actinomyces* spp.		8 (13%)		5 (13%)		3 (12%)		12 (22%)
*Capnocytophaga* spp.								1 (2%)
*Lachnoanaerobaculum orale*		1 (2%)				1 (4%)		9 (16%)
*Campylobacter* spp.		1 (2%)		1 (3%)				1 (2%)
*Dialister* spp.		2 (3%)		2 (5%)				
*Propionibacterium acnes*		1 (2%)				1 (4%)		
Anaerobes (unspecified)		3 (5%)		2 (5%)		1 (4%)		8 (15%)
**Fungi**								
Candida		5 (8%)		2 (5%)		3 (12%)		1 (2%)
Number of species, mean (SD)		6.5 (1.6)		6.4 (1.5)		6.7 (1.6)		8.3 (1.4)

*S. pyogenes* was significantly more prevalent in patients (27%) compared to controls (4%) (*p* < 0.001, Fisher´s exact test). The following species were significantly less frequently cultured in patients compared to controls: *H. parainfluenzae* (25% vs. 69%, *p* < 0.001), *Haemophilus* spp. (2% vs. 24%, *p* < 0.001), *Neisseria* spp. (64% vs. 96%, *p* < 0.001), *Fusobacterium* spp. (3% vs. 31%, *p* < 0.001), *Prevotella* spp. (83% vs. 100%, *p* < 0.001), *Leptotrichia* spp. (8% vs. 45%, *p* < 0.001), *Actinomyces odontolyticus* (17% vs. 36%, *p* = 0.022), and *Lachnoanaerobaculum orale* (2% vs. 16%, *p* = 0.006) (Table 2).

### Semi-quantitative analyses

Although, there was no significant difference in *F. necrophorum* between patients and controls (11% vs. 9%, *p* = 0.77) (Table 2), semi-quantitative culture findings showed significantly higher abundance of *F. necrophorum* in patients compared to the controls (2.4 vs. 1.4, *p* = 0.017, Kruskal Wallis-test). In addition, *S. pyogenes* was also cultured in significantly higher abundance in patients compared to controls (3.1 vs. 2.0, p = 0.045), while *Neisseria* spp. was isolated in significantly less abundance in patients than controls (2.4 vs. 2.6, *p* = 0.015) (Table 3).

**Table 3:** Semi-quantitative culture findings in tonsillar surface swabs from 64 patients with acute tonsillitis with or without peritonsillar phlegmon (PP) and 55 healthy controls. Means (SD) are given

	All n = 64	Controls n = 55	p^1^
Microorganisms			
**Aerobic bacteria**			
*Streptococcus pyogenes*	3.1 (0.7)	2.0 (0.0)	0.045
*Streptococcus dysgalactiae*	3.4 (1.0)	2.7 (0.6)	0.17
*Streptococcus anginosus* group	2.8 (1.1)	2.2 (0.4)	0.37
Non-hemolytic streptococci	3.4 (0.6)	3.3 (0.5)	0.43
*Haemophilus influenzae*	2.0 (----)	2.0 (----)	-
*Haemophilus parainfluenzae*	2.3 (0.4)	2.1 (0.3)	0.18
*Haemophilus* spp.	2.0 (----)	2.2 (0.4)	0.60
*Staphylococcus aureus*	2.4 (0.8)	2.3 (0.5)	0.94
Coagulase-negative staphylococci	2.2 (0.4)	2.0 (0.0)	0.44
*Eikenella corrodens*	2.4 (0.5)	2.0 (0.0)	0.33
*Neisseria* spp.	2.4 (0.7)	2.6 (0.6)	0.015
*Corynebacterium* spp.	2.3 (0.5)	2.2 (0.4)	0.15
*Rothia* spp.	2.9 (0.7)	2.7 (0.7)	0.20
*Aggregatibacter aphrophilus*		2.0 (----)	-
*Moraxella catarrhalis*	2.0 (----)		-
Enterobacteriaceae		2.0 (----)	-
*Gemella* spp.		2.0 (----)	-
*Granulicatella adiacens*	2.0 (0.0)		-
**Anaerobic bacteria**			
Fusobacterium necrophorum	2.4 (0.5)	1.4 (0.5)	0.017
*Fusobacterium* spp.	2.0 (0.0)	2.0 (0.0)	-
*Prevotella* spp.	2.8 (0.5)	2.8 (0.5)	0.94
*Veillonella* spp.	2.2 (0.4)	2.0 (0.2)	0.23
*Leptotrichia* spp.	2.2 (0.4)	2.0 (0.0)	0.49
*Lactobacillus* spp.	2.0 (0.0)	2.0 (0.0)	-
*Actinomyces odontolyticus*	2.5 (0.5)	2.4 (0.5)	0.47
*Actinomyces* spp.	2.5 (0.5)	2.3 (0.5)	0.30
*Capnocytophaga* spp.		2.0 (----)	-
*Lachnoanaerobaculum orale*	4.0 (----)	2.1 (0.3)	-
*Campylobacter* spp.	2.0 (----)	2.0 (----)	-
*Dialister* spp.	2.0 (0.0)		-
Anaerobes (unspecified)	2.0 (0.0)	2.0 (0.0)	-
**Fungi**			
Candida	2.0 (0.0)	2.0 (----)	-

Semi-quantitative count: 1: 1 colony, 2: 2–49 colonies, 3: 50–500 colonies, 4: >500 colonies

^1^ Kruskal-Wallis test

### Impact of antibiotic treatment prior to admission

No statistically significant difference in mean number of species cultured (6.3 vs. 6.8, *p* = 0.17, Student’s *t*-test) was found between patients with or without antibiotic treatment prior to admission. With the exception of *Haemophilus influenzae* (25% vs. 12%, *p* = 0.02, Fisher´s exact test), no statistically significant differences were found in culture rates among other species in patients with or without antibiotic treatment prior to admission (data not shown).

### Time of infection and follow up

Tonsillar swabs were obtained from 41 (64%) patients at follow up (mean 23 days after admission (range 11–56 days)). The clinical and biochemical characteristics of patients with and without follow up culture resembled each other (apart from the proportion of patients, who were referred because of pain, see Additional file 2 in supplementary). The mean number of species cultured were significantly lower at time of infection (6.5) compared to follow up (7.5) (*p* = 0.006, Student’s *t*-test), which was significantly lower compared to controls (8.3) (p = 0.006) (Table 4).

**Table 4:** Culture findings in tonsillar surface swabs from 41 patients with acute tonsillitis with or without peritonsillar phlegmon obtained at time of infection and at follow up (11–56 days later)

	Obtained from cultures at time of infection only		Obtained from cultures at time of infection and follow up		Obtained from follow up cultures only		p^1^
Microorganisms							
**Aerobic bacteria**							
*Streptococcus pyogenes*		7 (17%)		1 (2%)			0.016
*Streptococcus dysgalactiae*		7 (17%)					0.016
*Streptococcus anginosus* group		1 (2%)		1 (2%)		5 (12%)	0.22
Non-hemolytic streptococci				40 (98%)		1 (2%)	1.00
*Haemophilus influenzae*		1 (2%)					1.00
*Haemophilus parainfluenzae*		5 (12%)		7 (17%)		20 (49%)	0.004
*Haemophilus* spp.		1 (2%)				1 (2%)	1.00
*Staphylococcus aureus*		6 (15%)		5 (12%)		2 (5%)	0.29
Coagulase-negative staphylococci		2 (5%)		2 (5%)		6 (15%)	0.29
*Eikenella corrodens*		5 (12%)		1 (1%)		3 (7%)	0.73
*Neisseria* spp.		7 /17%)		18 (44%)		9 (22%)	0.80
*Corynebacterium* spp.		5 (12%)		23 (56%)		11 (27%)	0.21
*Rothia* spp.		1 (2%)		28 (68%)		12 (29%)	0.003
*Moraxella catarrhalis*		1 (2%)					1.00
*Actinetobacter* spp.						1 (2%)	1.00
*Pseudomonas* spp.						1 (2%)	1.00
*Gemella* spp.						3 (7%)	0.25
*Granulicatella adiacens*		3 (7%)					0.25
*Leuconostoc* spp.						1 (2%)	1.00
**Anaerobic bacteria**							
*Fusobacterium necrophorum*		4 (10%)					0.13
*Fusobacterium* spp.		1 (2%)				3 (7%)	0.63
*Prevotella* spp.		10 (24%)		25 (61%)		2 (5%)	0.039
*Veillonella* spp.		6 (15%)		12 (29%)		10 (24%)	0.45
*Leptotrichia* spp.		2 (5%)		2 (5%)		8 (20%)	0.11
*Lactobacillus* spp.		2 (5%)		4 (10%)		8 (20%)	0.11
*Actinomyces odontolyticus*		6 (15%)		2 (5%)		9 (22%)	0.61
*Actinomyces* spp.		5 (12%)		2 (5%)		7 (17%)	0.77
*Capnocytophaga* spp.						5 (12%)	0.063
*Lachnoanaerobaculum orale*						3 (7%)	0.25
*Campylobacter* spp.						1 (2%)	1.00
*Dialister* spp.		2 (5%)					0.50
Anaerobes (unspecified)						1 (2%)	1.00
*Parvimonas micra*						1 (2%)	1.00
*Propionibacterium acnes*		1 (2%)					1.00
**Fungi**							
Candida		2 (5%)					0.50
Number of species, mean (SD)		6.5 (1.7)				7.5 (1.6)	0.006

^1^ Binomial probability test (number at time of infection vs. at time of follow up), except for number of species (Student´s *t*-test)

The following species were significantly more prevalent in cultures at time of infection compared to follow up: *S. pyogenes* (17% vs. 0%, *p* = 0.016, binomial probability test), *S. dysgalactiae* (17% vs. 0%, *p* = 0.016), and *Prevotella* spp. (24% vs. 6%, *p* = 0.039). On the contrary, the following species were cultured significantly less frequent at time of infection compared to follow up: *H. parainfluenzae* (12% vs. 49%, *p* = 0.004) and *Rothia* spp. (2% vs. 29%, *p* = 0.003). *F. necrophorum*, *H. influenzae*, *Moraxella catarrhalis*, *Granulicatella adiacens*, *Dialister* spp., and Candida were found at time of infection only (Table 4).

No statistically significant differences in semi-quantitative abundance were found for the different species at time of infection compared to follow up (data not shown).

## Discussion

### Patient characteristics

The 64 patients included in the present study constitute a highly selected group of patients with severe AT with or without peritonsillar involvement, but without abscess formation, who were referred from general practice for specialized management by oto-rhino-laryngologists. The patients had substantial symptoms of AT, clear clinical signs of infection, and highly elevated C-reactive protein (mean: 167 mg/L) and neutrophil count (mean: 11.6 × 10^9^/L) suggestive of bacterial infection. Hence, the included patients had an urgent need for treatment and the suspicion of bacterial aetiology was very high. However, no prospective studies have been conducted exploring the significant pathogens in this group of patients. Based on tradition and the knowledge on the importance of *S. pyogenes* in AT, penicillin (benzyl- or phenoxymethyl-) is the standard antibiotic treatment for severe AT in Denmark.

### Suggested significant pathogens

Using meticulous and comprehensive culture methods, abundant polymicrobial aerobic and anaerobic flora was found in tonsillar swabs from AT patients as well as healthy controls. Based on the relative prevalence and semi-quantitative abundance between patients (at time of infection and follow up) and controls, pathogenic significance for the following bacteria was suggested: *S. pyogenes*, *F. necrophorum*, and *S. dysgalactiae*.

*S. pyogenes* was the only bacteria cultured with both significantly higher prevalence (17% vs. 4%) and semi-quantitative abundance (3.1 vs. 2.0) in patients compared to controls in addition to higher prevalence at time of infection than follow up (17% vs. 0%). These findings are in line with previous studies of patients with pharyngeal infections from uncomplicated AT [3] to PTA [13–15], and parapharyngeal abscesses [16]. The proportion of *S. pyogenes*-carriage in healthy individuals vary in different studies. In line with our findings, mean carriage-rates of 2.8% in adults and 8.0% in children were reported by Oliver et al. in a meta-analysis [17].

*F. necrophorum* was cultured in significantly higher abundance in patients compared to controls (2.4 vs. 1.4). The role of *F. necrophorum* in AT is unclarified [18], but more studies suggest that this anaerobe may play a role in uncomplicated cases. In PTA, *F. necrophorum* is established as the prevalent pathogen, while the importance in parapharyngeal abscesses is less certain [13, 15, 16].

In the current study, *S. dysgalactiae* was more prevalent at time of infection (17%), than at follow up (0%). A few previous studies have investigated the role of *S. dysgalactiae* in AT. A meta-analysis from 2020 to 11 studies suggested an association between *Streptococcus* group C (*S. dysgalactiae*) and uncomplicated sore throat in adults, even though it was much less evident than for *S. pyogenes* [19]. Lindbæk and colleagues investigated the role of *S. dysgalactiae* versus *S. pyogenes* in 306 patients with AT and found that 48% of the *S. pyogenes*-positive patients and 45% of the *S. dysgalactiae*-positive patients met three or four of the Centor criteria [20]. They concluded that *S. dysgalactiae* should be considered as a throat pathogen in line with *S. pyogenes.* In a study of 3,700 AT patients treated in primary health care, Nygren et al. found lower complication rates among patients positive for Group C/G streptococci (16%) compared to those positive for *S. pyogenes* (28%) and *F. necrophorum* (26%) [21].

*Prevotella* spp. was significantly more prevalent at time of infection (85%) compared to follow up (66%). However, this anaerobe was cultured from all controls (100%), suggesting that *Prevotella* spp. is part of commensal tonsillar flora and that the relatively low culture rate at follow up reflects the disturbed tonsillar flora at this time point. Based on these findings, we did not include *Prevotella* spp. among bacteria with suggested pathogenicity in AT. Brook and colleagues have conducted a number of serological studies showing increased antibody levels to *Prevotella intermedia* in patients with AT, peritonsillar cellulitis, infectious mononucleosis, PTA, and recurrent non-streptococcal tonsillitis [22–25]. More recently, our group conducted a prospective study of 60 patients with parapharyngeal abscesses [16]. *Prevotella* spp. was found in 12% of pus swabs, often in absolute and/or relative abundance and we concluded that this anaerobe was among four suggested significant pathogens.

In a similar, comparative study aimed to identify the significant pathogens in PTA, *S. pyogenes* and *F. necrophorum* were isolated significantly more frequently (both in tonsillar cores and surface swabs) among 36 patients than 80 controls, while Group C/G streptococci were found equally often [14].

Despite a meticulous and comprehensive culture and identification process, the three suggested pathogens in AT (*S. pyogenes*, *F. necrophorum* and *S. dysgalactiae*) were only detected in 48% of patients, leaving 52% of patients without suggested bacterial pathogens. Patients with no suggested pathogens were more frequently treated with antibiotics prior to admission (64% vs. 32%, *p* = 0.014, Fisher´s exact test), had longer duration of symptoms (median: 4.0 vs. 3.0 days, *p* = 0.034, Kruskal-Wallis test), and had lower (though still elevated) leukocyte and neutrophil counts (means: 12.1 vs. 17.1 and 9.2 vs. 13.9 × 10^9^/L, respectively, both p < 0.001) compared to patients with one of the three pathogens cultured. An interpretation could be that patients, who have had symptoms for longer were more likely to receive antibiotics, which in turn reduced the chance of culturing the three penicillin-susceptible pathogens in question. Another interpretation could be that patients without one of the three pathogens were infected with a virus or a less violent bacterium. The substantial symptoms and findings in addition to the elevated infection markers brings the authors to find the first interpretation most likely.

### Dysbacteriosis

The mean number of species cultured was significantly lower in patients at time of infection compared to controls (6.5 vs. 8.3) and the following species, belonging to the commensal pharyngeal flora, were detected significantly less frequently: *Prevotella* spp., *Rothia* spp., *Neisseria* spp., *H. parainfluenzae*, *Leptotrichia* spp., *Fusobacterium* spp., *Haemophilus* spp., and *Lachnoanaerobaculum orale*. After clinical recovery, the number of species cultured increased at follow up (mean 7.3) suggesting a trend towards normalization of the pharyngeal flora. Hence, infections were not only associated with increased presence of a few potential pathogens, but a reduced diversity of the pharyngeal flora (dysbacteriosis) was also evident. Dysbacteriosis is well-described in other human body sites where microorganisms (microbiota) live in communities on the skin surface or mucosal membrane (e.g. the oral cavity and the gastrointestinal tract). The composition of the microbiota is affected by the environment and antibiotic treatment, smoking, increasing age, and a number of diseases have been shown to cause alterations in the microbial composition and diversity [26, 27]. Whether the observed dysbacteriosis in the current study is secondary to AT or a factor paving the road for the invasive bacteria is speculative.

#### Previous studies on patients referred to hospital with acute tonsillitis

To our knowledge, the current study is the first to prospectively investigate the culturable microorganisms among patients with AT with or without PP referred for treatment in a hospital setting. A few previous retrospective studies have described the bacteriological findings in routine cultures with no further attempts to identify the significant pathogens [9–11]. Rusan et al. detected *S. pyogenes* (23%), *S. dysgalactiae* (11%), and *F. necrophorum* (5%) in 109 throat swabs from 327 patients [9]. Suzuki et al. included 127 AT patients from 36 Ear-Nose-Throat departments and clinics. Bacterial cultures from throat swabs showed; *S. pyogenes* (12%), *Staphylococcus aureus* (3%), *Streptococcus pneumonia* (2%), and *H. influenza* (1%) [11]. It was not stated whether patients were hospitalized or treated in an outpatient setting. Lastly, Risberg et al. detected *S. pyogenes* in 35 of 102 patients with PP using a rapid antigen detection test. No further bacterial investigations were made [10].

### Limitations

Our conclusions concerning the significant pathogens associated with severe AT should be interpreted in light of the relatively limited number of patients and controls (risk of type II errors). The study relies on bacterial cultivation and swabs were frozen before processing, which may affect the ability to culture some fastidious or fragile microorganisms. The following precautions were made to minimize processing bias: (1) samples were rapidly transferred to -80 °C after collection. (2) a broad range of aerobic and anaerobic plate media were used to attempt exhaustive cultivation. (3) the same very experienced technician processed all samples. In addition, MALDI-TOF MS was used for accurate identification. However, an exhaustive species distinction using cultured-based methods is difficult and some species may have been un- or under-detected [28, 29].

Medical students were used as healthy controls. Though their ages and gender-ratio matched the patients well, controls were less often smokers and may not represent a normative population. However, the swabs were obtained and processed in the same way as for patients and our findings (*S. pyogenes* 4%, *F. necrophorum* 9%, and *S. dysgalactiae* 5%*)* are similar to previously reported culture-based detection rates for the suggested pathogens (*S. pyogenes* 1.1–2.3%, *F. necrophorum* 3.8–9.4%, and *S. dysgalactiae* 3.1–5.5%) [6, 17–[18, 30]–31]. Studies using polymerase chain reaction (PCR) methodology have reported higher detection rates with low quantities of bacteria present in the samples [30, 32], which is in agreement with our finding of significantly less abundance (semi-quantitative count) of *S. pyogenes* and *F. necrophorum* in controls compared to patients.

Lastly, multiple comparisons were made in the study and it could be argued that correction for multiple testing is appropriate. Such correction was not applied as our conclusions concern three bacteria, which have previously been suggested as pathogens in AT and, thus, the uncorrected statistically significant findings are unlikely to be results of chance. However, the p-values should be interpreted in light of the multiple comparisons made.

## Conclusion

Our findings suggest that *S. pyogenes*, *F. necrophorum*, and *S. dysgalactiae* are significant pathogens in severe AT with or without PP. In addition, infections were associated with reduced diversity (dysbacteriosis). Based on the fact that all three suggested pathogens are sensitive to penicillin, we advocate the use of phenoxymethyl-penicillin or benzyl-penicillin for the treatment of patients, who are referred to hospital with AT or PP. Further investigations with non-culture-based methods such as whole genome sequencing or microbiome analyses may provide more detailed information on the causative microorganisms within this group of patients.

## Electronic supplementary material

Below is the link to the electronic supplementary material.

**Additional file 1**. Clinical and biochemical characteristics of 64 patients with or without follow up cultures. **Additional file 2**. Methods used for identification of bacteria.

## Data Availability

Anonymized data can be obtained from the corresponding author upon request.

## Abbreviations

AT: Acute tonsillitis
PP: Peritonsillar phlegmon
PTA: Peritonsillar abscess
MALDI-TOF MS: Matrix-assisted laser desorption/ionization time-of-flight mass spectrometry
PCR: Polymerase chain reaction
