# Supplementary material for: Bacterial findings in patients referred to hospital for the treatment of acute tonsillitis with or without peritonsillar phlegmon
Source: BMC Infect Dis. 2023 Jun 29;23:439. doi: 10.1186/s12879-023-08420-8 (PMC10308605; doi:10.1186/s12879-023-08420-8)
Supplement: Supplementary file 1 — Additional file 1. Clinical and biochemical characteristics of 64 patients with or without follow up cultures. Additional file 2. Methods used for identification of bacteria. [file 12879_2023_8420_MOESM1_ESM.docx]

| Additional file 1. Methods used for identification of bacteria. |
| --- |
| Identified based on phenotypic appearance:  Non-hemolytic streptococci Staphylococcus aureus Coagulase-negative staphylococci  *Neisseria spp.*  *Corynebacterium spp.*  Anaerobes (unspecified)  *Candida spp.* |
| Identified based on MALDI-TOF MS^1^:  *Streptococcus pyogenes*  *Streptococcus dysgalactiae*  *Streptococcus anginosus* group  *Haemophilus influenza*  *Haemophilus parainfluenzae* Haemophilus spp. *Eikenella corrodens*  *Rothia spp.* Moraxella catarrhalis *Acinetobacter spp.*  *Pseudomonas spp.*  *Gemella spp.*  *Granulicatelle spp.*  *Leuconostoc spp.*  *Fusobacterium necrophorum*  *Fusobacterium spp.*  *Prevotella spp.*  *Veillonella spp.*  *Leptotrichia spp.*  *Lactobacillus spp.*  *Actinomyces odontolyticus*  *Actinomyces spp.*  *Capnocytophaga spp.*  *Lachnoanaerobaculum orale*  *Campylobacter spp.*  *Dialister spp.*  *Parvimonas micra*  *Propionibacterium acnes*  *Aggregatibacter aphrophilus*  *Enterobacterales* |

^1^ Matrix-assisted laser desorption/ionization time-of-flight mass spectrometry

| Additional file 2. Clinical and biochemical characteristics of 64 patients with or without follow up cultures. | | | | |
| --- | --- | --- | --- | --- |
|  | Follow up culture | | |  |
|  | All  n=64 | Yes  n=41 | No  n=23 | p |
| Males | 24 (38%) | 13 (32%) | 11 (48%) | 0.28 |
| Age, mean (SD) | 25.7 (5.6) | 25.5 (5.9) | 26.0 (5.2) | 0.72 |
| Duration of symptoms, days  mean (SD) | 4.6 (2.7) | 5.0 (2.8) | 3.7 (2.3) | 0.06 |
| Reasons for referral |  |  |  |  |
| Suspected peritonsillar abscess | 48 (75%) | 29 (71%) | 19 (83%) | 0.37 |
| Dehydration | 14 (22%) | 10 (24%) | 4 (17%) | 0.75 |
| Pain | 23 (36%) | 20 (49%) | 3 (13%) | 0.012 |
| Other^1^ | 6 (9%) | 4 (10%) | 2 (9%) | 1.0 |
| Antibiotic treatment prior to admission | 32 (50%) | 24 (60%) | 8 (35%) | 0.12 |
| Tobacco smoking (current) | 26 (41%) | 15 (37%) | 11 (32%) | 0.43 |
| Temperature, mean ^o^C (SD) | 38.4 (0.8) | 38.3 (0.8) | 38.4 (0.9) | 0.97 |
| Biochemistry, mean (SD) |  |  |  |  |
| C-reactive protein, mg/L | 167 (113) | 175(120) | 152 (99) | 0.44 |
| Leukocyte count, x10^9^/L | 14.6 (5.0) | 14.7 (4.7) | 14.5 (5.6) | 0.91 |
| Neutrophil count, x10^9^/L | 11.6 (4.7) | 11.5 (4.5) | 11.6 (5.1) | 0.97 |
| Lymphocyte count, x10^9^/L | 1.6 (0.7) | 1.7 (0.7) | 1.4 (0.6) | 0.15 |

^1^ Dyspnoea/upper airway obstruction (n=3), General malaise (n=1), Neck swelling (n=1), Lack of improvement after antibiotic treatment (n=1).
